# Profil épidémiologique et anatomopathologique du cancer de sein au CHU Ibn Rochd, Casablanca

**DOI:** 10.11604/pamj.2020.37.41.21336

**Published:** 2020-09-09

**Authors:** Majdouline El Fouhi, Abdellatif Benider, Kagambega Zoewendbem Arsène Gaëtan, Abdelhalim Mesfioui

**Affiliations:** 1 Laboratoire de Génétique-Neuro-Endocrinologie-Biotechnologie, Faculté des Sciences, Université Ibn Tofail, Kénitra, Maroc,; 2 Radiothérapie Oncologique, Centre Mohamed VI pour le Traitement des Cancers, CHU Ibn Rochd, Casablanca, Maroc,; 3 Service de Cancérologie, CHU de Treichville, Abidjan, Côte d’Ivoire

**Keywords:** Cancer, sein, épidémiologie, histologie, Maroc, Cancer, breast, epidemiology, histology, Morocco

## Abstract

Réaliser une revue récente comportant les particularités épidémiologiques, histologiques des patients nouvellement diagnostiqués d’un cancer de sein à Casablanca durant l’année 2018. Nous avons colligé 668 cas pendant l’année 2018, l’âge moyen était 51,6 ans, le sexe féminin était le plus représenté avec 662 cas (99,1%) et les hommes avec 6 cas (0,9%), soit un sexe ratio (H/F) de 0,009. L’âge moyen de la ménopause était 49,8 ans et l’âge moyen de la ménarche était 13,5 ans, 31,7% avait un antécédent de cancer (le sein 14,1%, l’estomac 9% et le foie 7%). Le délai moyen de consultation était 10 mois, la pathologie la plus représentée était la pathologie thyroïdienne. Selon la localisation, le sein gauche était atteint dans 50,2% et le sein droit dans 44,7% et une localisation bilatérale 1,3%, le siège le plus fréquent était le quadrant supérieur externe avec 28,7%, les stades T1 et T2 représentaient 73,6% et les stades T3 et T4 représentaient 26,4%, les tumeurs épithéliales infiltrantes carcinome canalaire infiltrant (CCI) étaient les plus fréquentes (73,2%). La présence des emboles vasculaires et lymphatiques a été notée chez 42,2%, les ganglions axillaires étaient atteints chez 71,1% des patients. Le grade histopronostique de Scarff Bloom et Richardson (SBR) avait révélé une prédominance du grade II dans 55,9% des cas. Le Luminal B continue à constituer le phénotype le plus fréquent (46%) suivi du Triple Negatif (15,3%) et du Luminal A (14,2%) et enfin le HER2 (7,4%). Le pronostic immédiat demeure inquiétant du fait du retard de diagnostic. Il nous paraît urgent de mettre en place une politique sanitaire d’information et d’éducation.

## Abstract (en)

The present study aims to determine the various epidemiological characteristics among newly diagnosed patients with breast cancer in Casablanca during 2018. During that period, 668 cases were collected, the average age was 51.6 years, the female was the most represented with 662 cases (99.1%) and men with 6 cases (0.9%), a sex ratio (M/F) of 0.009. The average age of menopause was 49.8 years and the average age of menarche was 13.5 years, 31.7% had a history of cancer (breast 14.1%, stomach and 9% liver 7%). The average diagnosis delay was 10 months, the thyroid disease was the most represented pathology, the left breast was diagnosed in 50.2% and the right breast in 44.7% and 1.3% in the bilateral location. The most common histological type was invasive ductal carcinoma (73.2%). The vascular and lymphatic invasion was observed in 42.2%, axillary nodes were affected in 71.1% of cases. The histological prognosis (SBR) revealed a predominance of grade II in 55.9% of cases. The Luminal B continues to be the most common phenotype (46%) followed by Triple Negative (15.3%) and Luminal A (14.2%) and HER2 (7.4%). The immediate prognosis is a cause for concern because of delayed diagnosis. It seems urgent to develop the health information policy and education.

## Introduction

Le cancer est un problème majeur de santé publique, selon l’organisation mondiale de la santé il constitue la deuxième cause de décès dans le monde à l’origine de 8,8 millions de décès en 2015. Près d’un décès sur 6 dans le monde est dû au cancer. Au Maroc, la localisation la plus fréquente, en considérant les deux sexes, était le cancer du sein qui occupait le premier rang et qui représentait 19,2%, suivi par le cancer du poumon 12,3% et le cancer colorectal 7,8% [1]. Le cancer du sein représentait 20% de tous les cancers enregistrés chez les deux sexes et 35,8% des cas enregistrés chez les femmes. La quasi-totalité des patients atteints était de sexe féminin avec une proportion de 99,1%. Le sexe masculin représentait moins de 1% des cas enregistrés [2]. Plusieurs facteurs de risque d’apparition du cancer du sein sont reconnus, tels que les antécédents familiaux du cancer du sein, l’âge avancé, la puberté précoce, la ménopause tardive, la nulliparité et l’obésité, mais aucun facteur n’a pu être impliqué directement à sa survenu, à l’exception de la transmission héréditaire du gène BRCA 1 et 2 qui est impliqué dans 5-10% de cas de cancer du sein, depuis la découverte de Bittner; nombreux virus sont soupçonnés dans l’étiologie du cancer du sein [3]. Le but de ce travail est de souligner à travers une étude rétrospective portant sur 668 cas colligées durant l’année 2018 au sein du service de l’oncologie au CHU IBN ROCHD de Casablanca; les particularités épidémiologiques, cliniques, histologiques des cancers de sein.

## Méthodes

Il s’agit d’une étude rétrospective descriptive. Les cas sont des patients nouvellement recrus au sein du service Mohammed VI pour le traitement des cancers au CHU IBN ROCHD de Casablanca, durant l’année 2018, on a inclus tous les patients atteints de cancer du sein y compris le sexe masculin. Toutes les tumeurs mammaires malignes confirmées histologiquement sans préjuger de leur type histologique; on a exclu les patients déjà suivi avant l’année 2018 et les tumeurs mammaires sans preuve histologique et les malades dont le dossier est vide. Les variables étudiées: l’âge, la parité, l’âge de ménopause et de la menarche, les pathologies associées au cancer de sein, les antécédents personnel d’un cancer, les circonstances de découverte, le délai de consultation, le grade SBR, la localisation, le stade TNM, le phénotype moléculaire, la localisation des métastases, l’atteinte ganglionnaire. Les données ont été recueillies à travers une consultation des dossiers d’hospitalisation au sein des archives sur une fiche pré établie et la saisie des textes et des tableaux a été faite à l’aide du logiciel EXCEL, XP et l’analyse des données ont été réalisée à l’aide du SPSS version 21.

## Résultats

**Figure 1:**
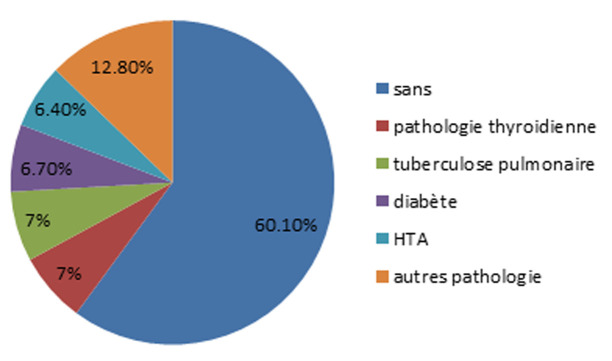
les pathologies les plus représentées chez les patients atteints de cancer de sein

**Figure 2:**
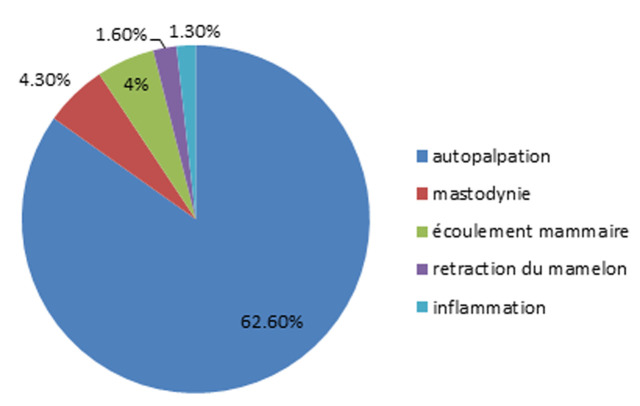
les circonstances de découverte du cancer de sein

Six cent soixante-huit cas de cancer de sein ont été diagnostiqué, pour la population féminine l’âge moyen était 51,6 ans avec des extrêmes de (23-89) ans et pour le sexe masculin l’âge moyen était 71 ans avec des extrêmes de (52-85) ans, 98,4% des patients étaient de sexe féminin et 0,9% étaient de sexe masculin, 21,1% des patients étaient nullipares, l’âge moyen de la ménopause était 49,8 ans avec des extrêmes de (24-63) ans et l’âge moyen de la ménarche était 13,5 ans avec des extrêmes de (10-17) ans. La pathologie la plus représentée était la pathologie thyroïdienne (Figure 1). Quatre-vingt-huit pourcent n’avait pas d’antécédent personnel d’une pathologie mammaire, 9% du reste avait subi une tumorectomie, 4% des patients avait un nodule bénigne. Trente et un virgule sept pourcent avait un antécédent de cancer dont le sein représentait 14,1% et l’estomac représentait 9% et le foie représentait 7%. L’autopalpation des seins était le moyen le plus représenté de découverte du cancer (Figure 2), le délai moyen de consultation était 10 mois avec des extrêmes de 1 mois et 132 mois (11 ans), 84,9% étaient sous couverture sociale dont 81,2% étaient des ramédistes.

**Figure 3:**
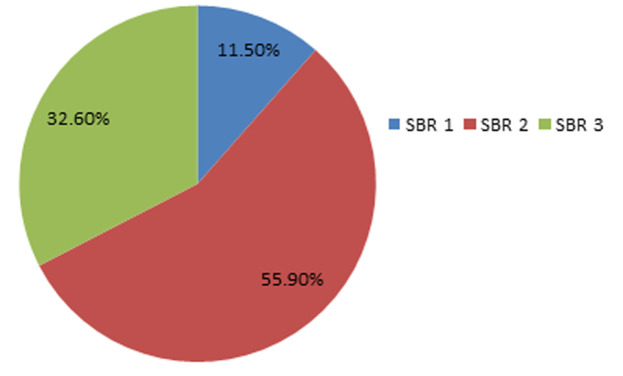
le grade SBR

**Figure 4:**
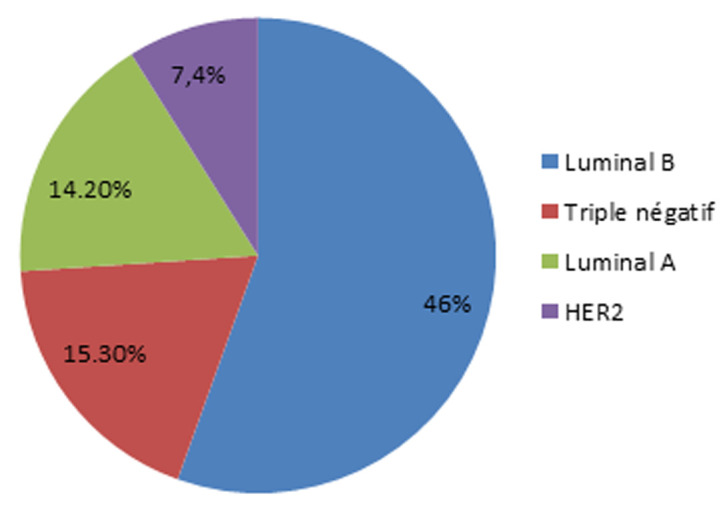
la distribution des phénotypes moléculaires

**Figure 5:**
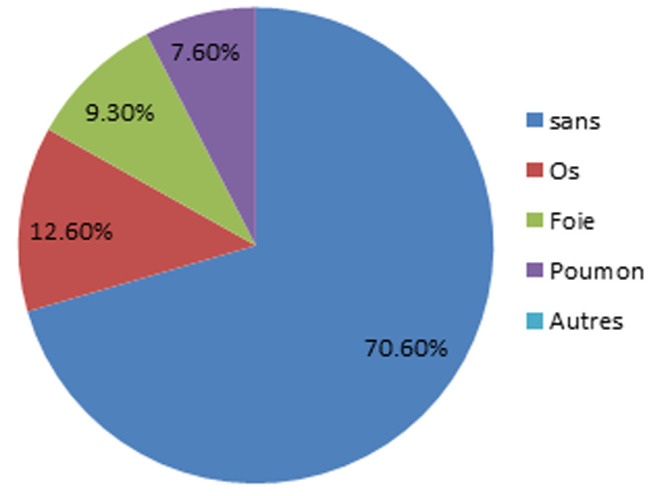
la localisation des métastases du cancer de sein

Le grade histopronostique de Scarff Bloom et Richardson (SBR 2) était le plus représenté dans notre série (Figure 3), dans notre série la taille tumorale clinique la plus marquée selon la classification TNM était le T1 et T2 avec un pourcentage de 73,6% (Tableau 1), le côté gauche était le fréquemment concerné avec 50,2% suivi par le sein droit (44,7%) et la localisation bilatérale (1,3%), 13,5% des patients avaient une mastopathie associé (fibrokystique 8,2%, sclérokystique 2,4%, mastite 2,1%, mastose 0,6%, galactophorite 0,1%), le siège le plus fréquent était le quadrant supérieur externe (QSE) avec 28,7%, 88,9% des patients présentaient un foyer unifocal et 8,5% présentaient un foyer bifocal et 2,6% présentaient un foyer multifocal. Le type histologique le plus fréquent était le carcinome canalaire infiltrant 73,2% des cas (Tableau 2). Pour le profil immunohistochimique, l’analyse a révélé que 46% étaient de type luminal B (Figure 4), l’immunohistochimie a été réalisé chez 568 des patients, la positivité des récepteurs oestrogéniques était marquée dans 55,7% des cas par contre les récepteurs progestéroniques qui n’était notée que chez 44,1% des cas. Vingt-neuf virgule cinq pourcent de nos patients ont présenté des métastases au moment de diagnostic, la localisation la plus fréquente était l’os (Figure 5), la présence des emboles vasculaires et lymphatiques a été notée chez 42,2%, les ganglions axillaires étaient atteints chez 71,1% des patients, la rupture capsulaire a été noté chez 22% des patients.

**Tableau 1:** la taille tumorale clinique selon la classification TNM

STADE	POURCENTAGE
T1 et T2	73,6%
T3 et T4	26,4%

**Tableau 2:** répartition des types histologique

Type histologique	Pourcentage
CCI	73,20%
Indifférencié	6,30%
CLI	6%
Mucineux	2,70%
Micropapillaire	2,24
Papillaire	2,04%
Médulaire	1,80%
Maladie de Paget du mamelon	1,30%
Carcinome mixte	1,20%
Tubuleux	0,14%
Aporicine	0,14%
Carcinome inflammatoire	0,14%
Phyllode	0,80%
Carcinome canalaire *in situ*	1,9%
Carcinome lobulaire *in situ*	0,1%

## Discussion

Nous avons colligé au total 668 cas de cancer du sein nouvellement diagnostiqués au cours de l’année 2018. Cette fréquence est plus élevée par rapport à celle trouvée en France et en Algérie et la Tunisie [1], au Maroc le sein présente 19,2% de l’ensemble des cancers [2]. Parmi les facteurs qui expliquent cette hausse de l’incidence des cancers: l'adoption du mode vie occidental au plan mondial (mauvaises habitudes alimentaires, sédentarité, obésité, le tabac...) La tranche d’âge 40-50 ans concentre la majorité des femmes affectées par cette pathologie. L’âge médian au moment du diagnostic de ce cancer était 51,6 ans. Ceci ne concorde pas avec les données obtenues en France (l’âge moyen: 61 ans, tranche d’âge: 60-69 ans) [1], mais nous rejoignons les résultats obtenus en Algérie (l’âge moyen: 50 ans, tranche d’âge: 50-54 ans) [4]. L’âge moyen à la ménopause est 49,8 ans et celui de la ménarche est 13,5 ans; il y’a une similarité avec l’étude faite en 2016 au même centre [5]. Les données de la littérature sont concordantes avec celles de notre étude: nous avons trouvé plus que la moitié de nos patientes ont eu leurs premières règles avant 12 ans, la puberté avant 12 ans augmente le risque de cancer du sein à l’âge adulte par une exposition plus prolongée aux œstrogènes. Le risque lié à la nulliparité est faible, dans notre étude; 21,1% des patientes étaient nullipares, nous rejoignons les résultats qui ont été trouvé précédemment: Mesmoudi (20,5%) [6], Benidar (17%) [7], Menikhar (29,6%) [8].

Un âge avancé à la ménopause ressort souvent aussi comme facteur de risque de développer un cancer du sein [9]. L’étude [9] retrouve pour un risque relatif de 1 pour les femmes ménopausées avant 45 ans, un risque relatif de 2,1 pour celles qui le sont après 55 ans. Dans notre série 37,7% des femmes étaient ménopausées, l’âge moyen de la ménopause était 50 ans. Dans notre étude, les femmes représentaient 97,60% des cas, soit un sexe ratio (H/F) de 0,02, donc on confirme ce qui a été décrit dans la littérature. La rareté des tumeurs du sein dans le sexe masculin s’explique par le caractère atrophique de la glande, la finesse des canaux galactophores, l’absence d’acini et l’abondance du tissu fibreux chez l’homme. Quatorze virgule un pourcent de nos patients avaient un antécédent familial d’un cancer, le cancer du sein est héréditaire dans 5 à 10% des cas. L’estimation du risque familial et individuel peut être un apport déterminant à la prise en charge de ces patients par la pratique de dépistage ou d’une prévention adaptée [10]. Douze pourcent avait un antécédent personnel d’un cancer de sein, l’existence d’un antécédent personnel de cancer du sein traité et guéri constitue également un facteur de risque de même qu’un antécédent personnel de cancer de l’ovaire ou du colon [11].

Les diagnostics interviennent souvent à un stade tardif, dans notre étude le délai moyen de consultation est de 10 mois, cela pourrait être dû à une insuffisance de l’éducation pour la santé et les conditions socio-économiques médiocres des populations, presque 60% de nos patients étaient dans un stade métastatique au moment de diagnostic, en tenant compte de tous ces éléments, il est évident de se lancer et renforcer les campagnes de dépistage et de sensibilisation pour résoudre tous les problèmes. Soixante-dix-huit virgule deux pourcent des patients révélaient la maladie par l’autopalpation d’un nodule suivi par des mastodynies dans 6% des cas. Dans 56,32% des cas la tumeur intéressait le sein gauche. La prédominance du cancer au niveau d’un sein par rapport à l’autre s’explique par les habitudes d’allaitement [12]. Dans la littérature, le cancer du sein est généralement unilatéral et un peu plus souvent dans le côté gauche, il atteint rarement les deux seins. C’est ce que confirme notre étude, le sein gauche présentait 50,2% suivi pas le sein droit (44,7%) et la localisation bilatérale (1,3%), la tumeur a été retrouvé au QSE dans 28,7% des cas, au QSI dans 7,3%, indépendamment du côté du sein atteint. La localisation rétromamellonnaire quant à elle n'a été présenté que chez 4,19% des cas, on note toujours une domination du siège QSE ainsi qu’à travers plusieurs études [13] cette topographie s’explique par la quantité du tissu glandulaire toujours plus présente dans la partie centrale et supero-externe du sein [14,15].

Un nombre relativement élevé de malades jeunes pose, en matière de prise en charge, des problèmes supplémentaires. En effet, plusieurs travaux [16-19] ont signalé que le cancer du sein chez la femme jeune est plus souvent agressif avec une fréquence plus élevée de grade 3 de la classification SBR, et de récepteurs ostrogéniques négatifs, dans notre étude 18,7% des patients étaient jeunes (un âge <40 ans). La mesure de la taille tumorale aussi bien clinique que macroscopique constitue un important élément pronostic nécessaire à la prise en charge thérapeutique. Dans notre série on remarque une légère diminution des formes avancées T3 et T4 par rapport aux résultats trouvés dans les études précédentes Mesmoudi [8] et Marrakech [20]. Le type histologique a été précisé chez 663 patients, les tumeurs épithéliales infiltrantes étaient les plus fréquentes avec une prédominance du carcinome canalaire infiltrant 73,2%, ces pourcentages restent stables par rapport aux résultats obtenus dans les années précédentes. Plusieurs études ont montré une corrélation positive entre la multifocalité et la présence de métastases axillaires [21]. La multifocalité a été observé chez 11% de nos patients et 35% de ces patients ont eu des métastases ganglionnaires.

De nombreuses études ont établi que les patients présentant des métastases locorégionales ont un moins bon pronostic que celles ne présentant pas d’envahissement ganglionnaire. De manière globale, la survie à dix ans est de 70% quand il n’y a pas d’atteinte ganglionnaire et est de 25 à 30% en présence d’envahissement néoplasique des ganglions [22]. Dans notre série 35,7% présentaient des envahissements ganglionnaires, et une moyenne de 3 ganglions qui sont envahis. Toutes les études montrent que le risque métastatique et la survie sont fortement corrélés au grade, quel que soit le système de grading utilisé, ainsi le grade III est de mauvais pronostic par rapport au grade I et II. Le grade histopronostic de SBR a été étudié chez 621 patients dans cette série et il a révélé la prédominance du grade II avec un pourcentage de 60%, suivi du grade III (20%). Ce classement a également été celui de l’étude faite en 2016 dans le même centre [23]. Les récepteurs hormonaux aux œstrogènes sont des marqueurs de différenciation tumorale alors que la positivité des récepteurs aux progestérones témoigne de la fonctionnalité des récepteurs aux œstrogènes. Les récepteurs hormonaux aux progestérones sont positifs dans 40 à 50% des cas, ce sont des facteurs pronostiques puisque l’expression de ces récepteurs est un élément de bon pronostic et surtout prédictif de la réponse au traitement hormonal [24]. Les récepteurs hormonaux ont été étudié chez 568 patients, ces récepteurs ont été positif chez 60,7% des bilans réalisés.

## Conclusion

Le diagnostic tardif continu à aggraver le pronostic de ce cancer, les tumeurs dont le diamètre est de 5cm constituent un fort pourcentage, où la nécessité d’œuvrer pour baisser ce pourcentage. Les autres aspects: épidémiologiques, cliniques et histopathologiques présentent les mêmes particularités que les résultats de la littérature des pays en développement. A travers cette étude nous concluons les points suivants: 1) le cancer du sein occupe, dans notre série, la première place par rapport aux autres cancers gynéco-mammaires. 2) La découverte par examen médical reste une éventualité rare (2,2%). 3) Le délai de consultation tardif (10 mois en moyenne). 4) Le taux des tumeurs diagnostiquées à un stade tardif reste relativement important. 5) Le taux des tumeurs avec grade histopronostique élevé est important (SBR II et SBR III: 88,5%) et l’envahissement ganglionnaire histologique intéressait 35,7% des cas. 6) Une domination des tumeurs épithéliales infiltrantes (CCI 73,2%). Le cancer de sein reste une pathologie grave difficile à surmonter, sa prise en charge reste entravée par les conditions socio-économiques, ce qui nécessite une mise en place de politique de dépistage à coût abordable par la population et une poursuite des campagnes de sensibilisation.

### Etat des connaissances sur le sujet

- Le cancer de sein est le cancer le plus fréquent chez les femmes;
- Il est en particulier de plus en plus fréquent dans les pays en développement où la majorité des cas sont diagnostiqués à des stades avancés;
- Un dépistage précoce reste le principal moyen de lutter contre la maladie.

### Contribution de notre étude à la connaissance

- Le cancer du sein continue à occuper la première place par rapport aux autres cancers gynéco-mammaires;
- Le délai de consultation reste toujours tardif (10mois), ce qui rend le pronostic mauvais;
- Une domination de la forme infiltrante des tumeurs et une légère diminution des formes avancées T3 et T4.
